# Supplementary material for: Propagating wave in a fluid by coherent motion of 2D colloids
Source: Nat Commun. 2021 Nov 19;12:6771. doi: 10.1038/s41467-021-26917-1 (PMC8605016; doi:10.1038/s41467-021-26917-1)
Supplement: Supplementary file 1 — Supplementary Information file [file 41467_2021_26917_MOESM1_ESM.pdf]

Supplementary Information

Propagating wave in a fluid by coherent motion of 2D colloids

Koki Sano[1,2], Xiang Wang[1], Zhifang Sun[1], Satoshi Aya[1], Fumito Araoka[1],

Yasuo Ebina[3], Takayoshi Sasaki[3], Yasuhiro Ishida[1]* and Takuzo Aida[1,4]*

[1] RIKEN Center for Emergent Matter Science, 2-1 Hirosawa, Wako, Saitama 351-0198, Japan.

[2] JST PRESTO, 4-1-8 Honcho, Kawaguchi, Saitama 332-0012, Japan.

[3] National Institute for Materials Science, International Center for Materials Nanoarchitectonics, 1-1 Namiki, Tsukuba, Ibaraki 305-0044, Japan.

[4] Department of Chemistry and Biotechnology, School of Engineering, The University of Tokyo, 7-3-1 Hongo, Bunkyo-ku, Tokyo 113-8656, Japan.

*To whom correspondence should be addressed.

E-mail: y-ishida@riken.jp (Y.I.); aida@macro.t.u-tokyo.ac.jp (T.A.)

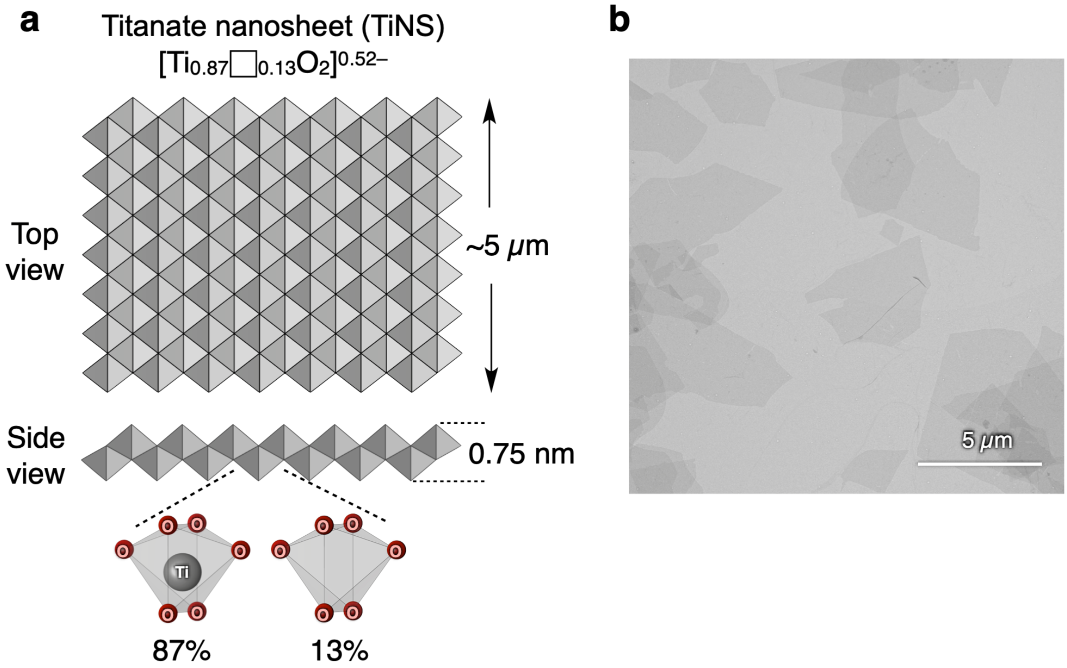

Supplementary Fig. 1 | Structure of titanate nanosheet (TiNS).

a, Schematic illustration of unilamellar titanate nanosheet (TiNS). Counterions $(CH_3)_4N^+$ are omitted for clarity. □: vacant site. **b**, Transmission electron microscopy (TEM) image of TiNSs deposited on a hydrophilized carbon-covered copper grid. From TEM images taken under similar conditions, the average lateral size of TiNSs is estimated to ~5 μm.

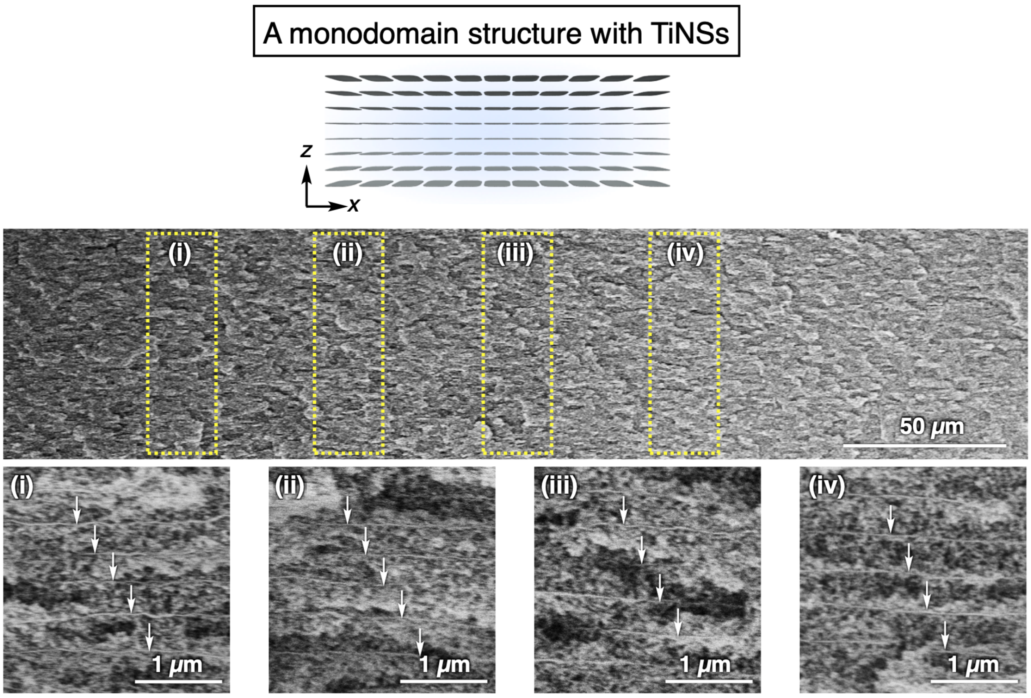

Supplementary Fig. 2 | Scanning electron microscopy (SEM) images of the monodomain structure with TiNSs.

The monodomain structure with TiNSs, formed in their aqueous dispersion ([TiNS] = 0.5 wt%) in a quartz cuvette (40 × 10 × 1 mm) at 25 °C under a 10 T magnetic field, was fixed by two-stage in-situ polymerization of an acrylic monomer and a silica source, then dried, and subjected to longitudinal-sectional SEM analysis.

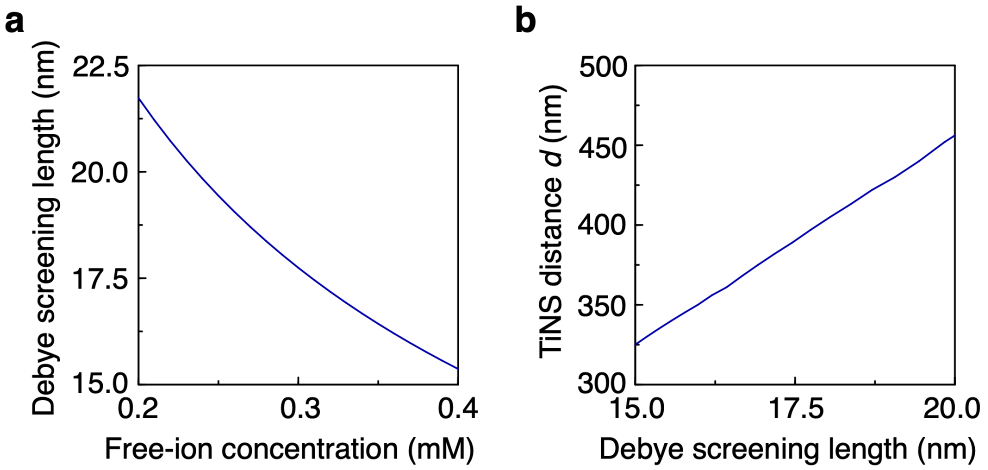

Supplementary Fig. 3 | Relationship between free-ion concentration, Debye screening length, and TiNS distance.

Plots of the Debye screening length against the free-ion concentration (**a**) and the TiNS distance against to the Debye screening length (**b**). For each data point of the free-ion concentration, the Debye screening length and TiNS distance were calculated by using the DLVO theory.

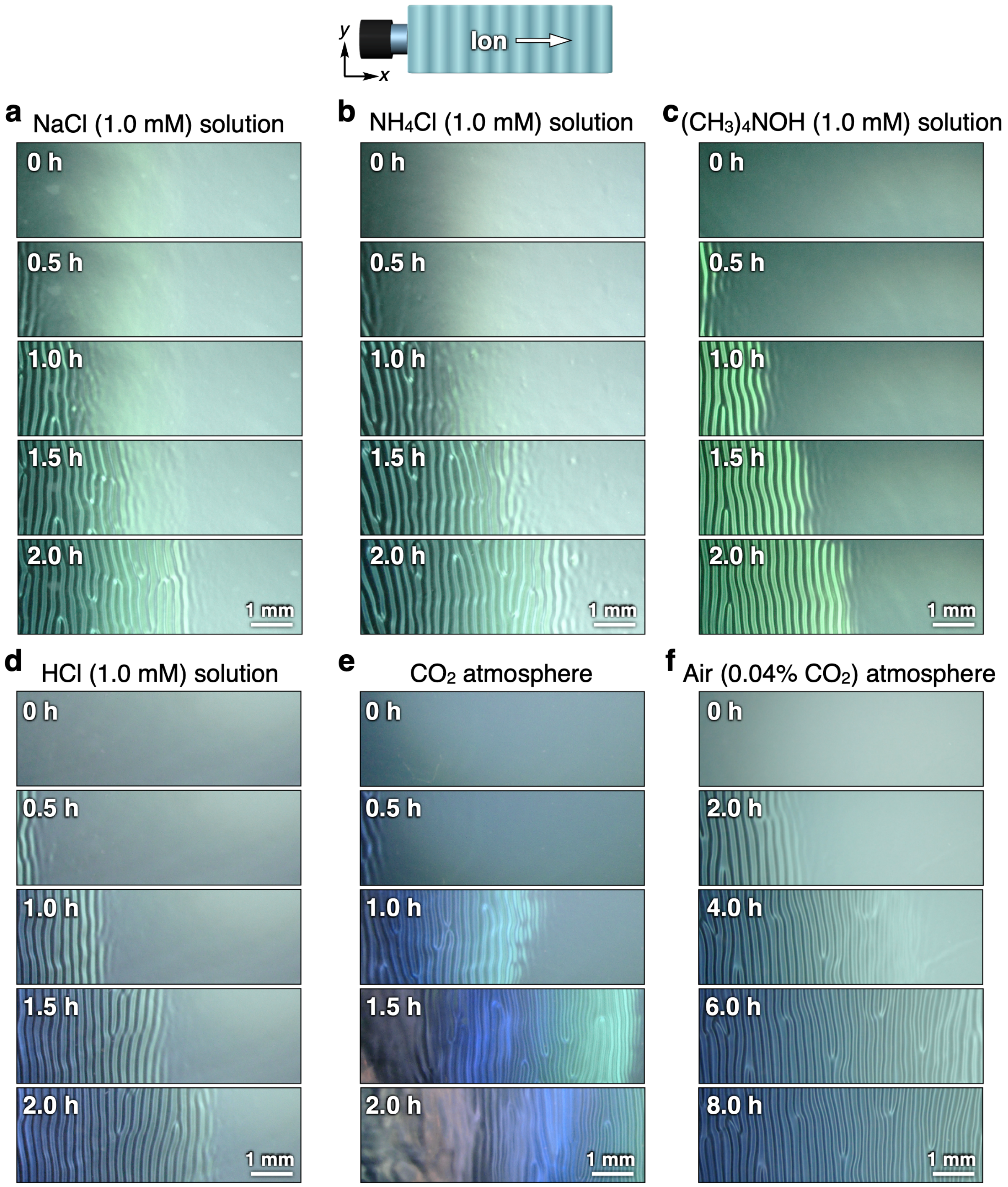

Supplementary Fig. 4 | Wave propagation by using different ionic species.

Time-dependent optical images of magnetically oriented TiNS dispersions ([TiNS] = 0.5 wt%) in quartz cuvettes (40 × 10 × 1 mm) at 25 °C that were treated with 1.0 mM aqueous solutions of NaCl (**a**), NH_4Cl (**b**), $(CH_3)_4NOH$ (**c**), HCl (**d**), CO_2 atmosphere (**e**), and air (0.04% CO_2) atmosphere (**f**) after turning off the magnetic field.

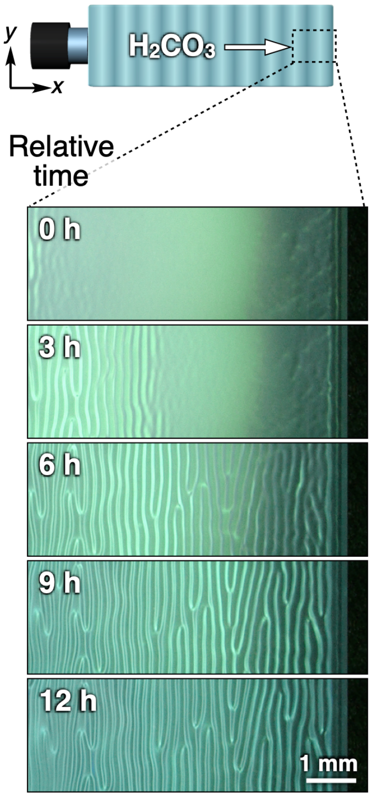

Supplementary Fig. 5 | Wave propagation near the closed end of a cuvette.

Time-dependent optical images of a magnetically oriented TiNS dispersion ([TiNS] = 0.5 wt%) at a region near the closed end of a quartz cuvette (40 × 10 × 1 mm) at 25 °C in air (0.04% CO_2) after turning off the magnetic field.

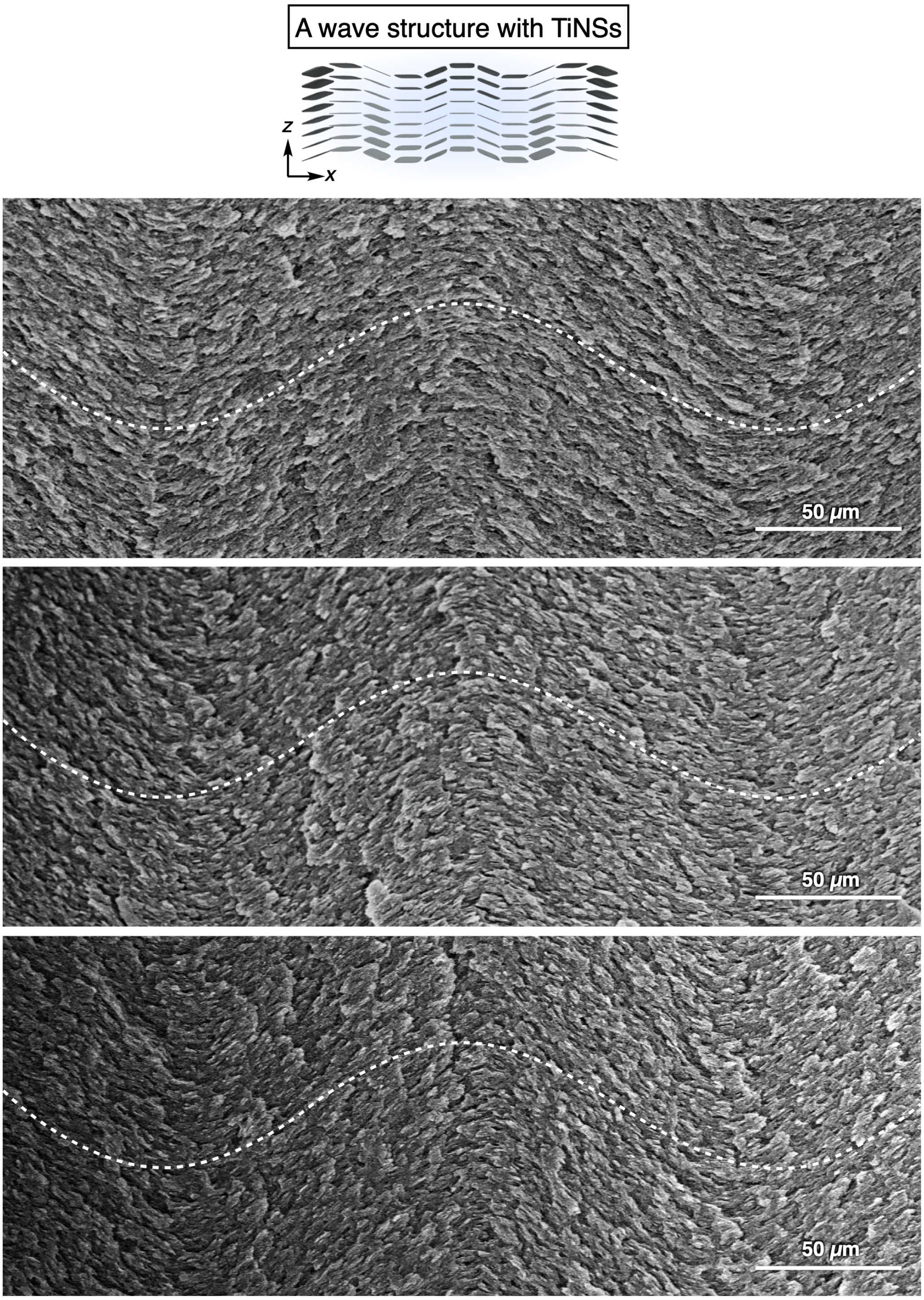

Supplementary Fig. 6 | Scanning electron microscopy (SEM) images of the wave structure with TiNSs at three different regions.

The wave structure with TiNSs, formed in their aqueous dispersion ([TiNS] = 0.5 wt%) in a quartz cuvette (40 × 10 × 1 mm) at 25 °C in air (0.04% CO_2), was fixed by two-stage in-situ polymerization of an acrylic monomer and a silica source, then dried, and subjected to longitudinal-sectional SEM analysis. Dashed white curves indicate the TiNS orientation.

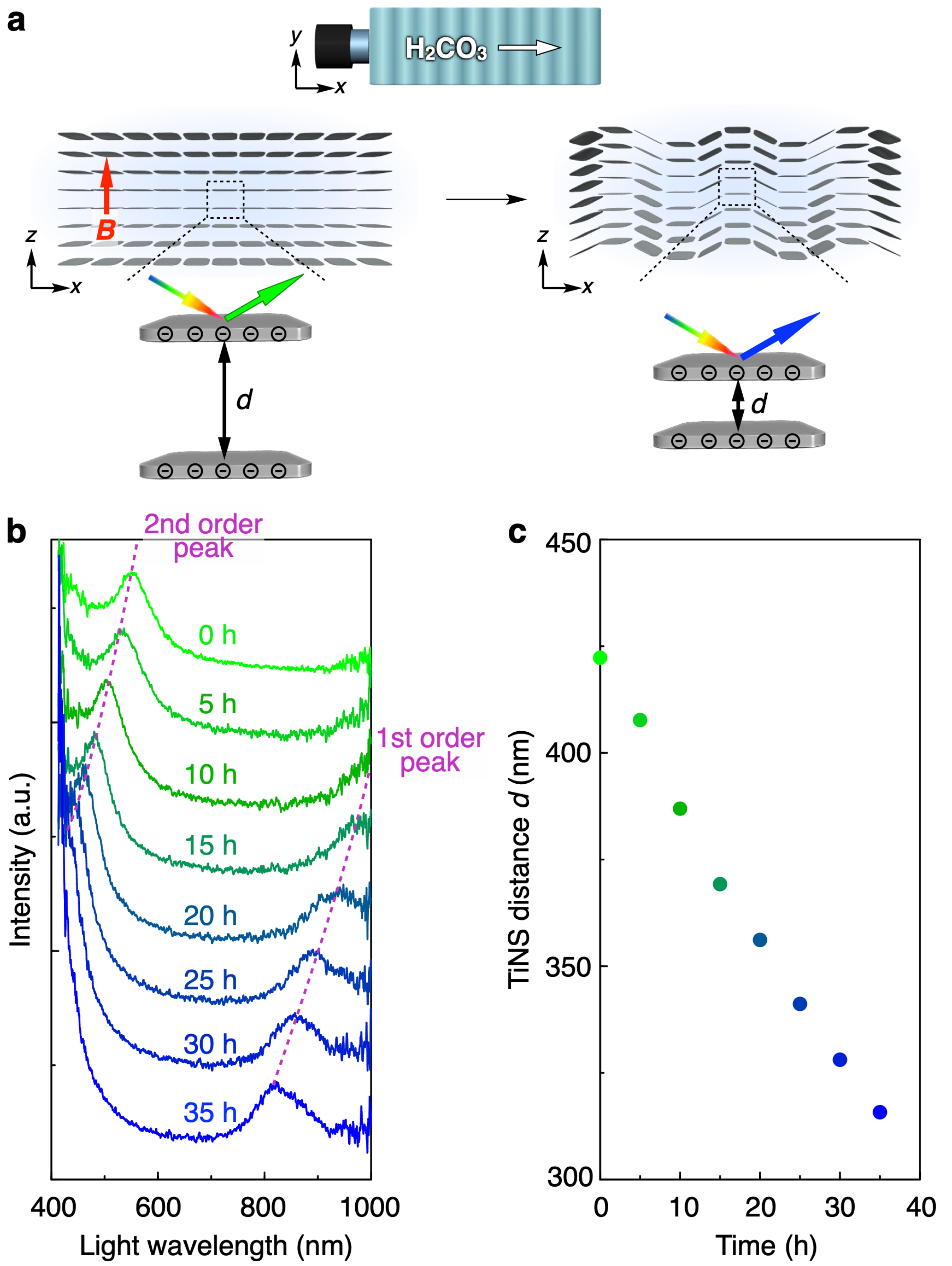

Supplementary Fig. 7 | Time-dependent changes of the TiNS distance.

a, Schematic illustrations of the TiNS distance changes accompanying structural color changes of the TiNS dispersion. **b**, Time-dependent changes of the microscopic reflection spectrum at a fixed distance *x*. **c**, Time-dependent changes in the TiNS distance calculated by using the Bragg's law. In **b** and **c**, a magnetically oriented TiNS dispersion ([TiNS] = 0.5 wt%) in a quartz cuvette (40 × 10 × 1 mm) was monitored at 25 °C in air (0.04% CO_2) after turning off the magnetic field.

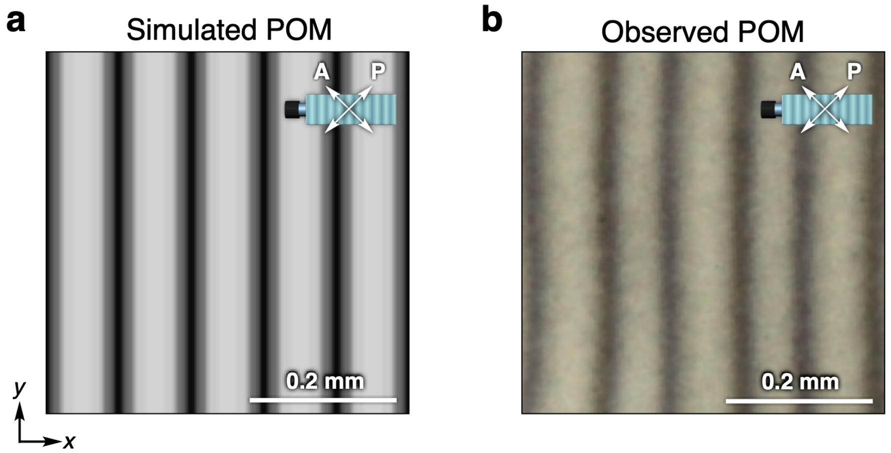

Supplementary Fig. 8 | Simulated and observed polarized optical microscopy (POM) images.
a, POM image simulated by using the Jones matrix method for the theoretically calculated wave structure in Fig. 2d. **b**, POM image experimentally observed under crossed Nicols of the wave structures generated in a magnetically oriented TiNS dispersion ([TiNS] = 0.5 wt%) in a quartz cuvette (40 × 10 × 1 mm) at 25 °C in air (0.04% CO_2) 24 h after turning off the magnetic field.

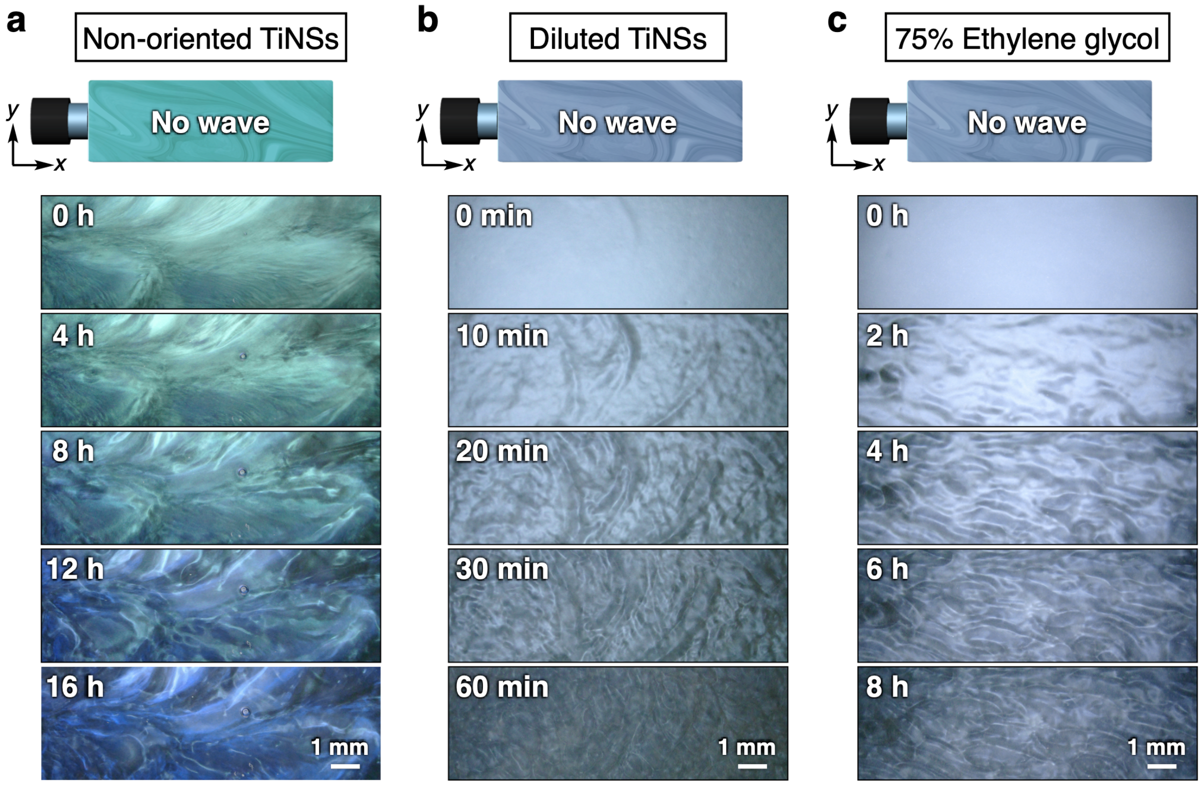

Supplementary Fig. 9 | Time-dependent behavior of TiNS dispersions without orientation and strong correlation.

a–c, Time-dependent optical images of a non-oriented TiNS dispersion ([TiNS] = 0.5 wt%; **a**), a magnetically oriented TiNS dispersion with a low concentration ([TiNS] = 0.2 wt%; **b**), and a magnetically oriented TiNS dispersion ([TiNS] = 0.5 wt%) in a low-permittivity medium (ethylene glycol / water [75:25, v/v]; **c**) in a quartz cuvette (40 × 10 × 1 mm) at 25 °C in air (0.04% CO_2). In **b** and **c**, the behaviors were monitored after turning off the magnetic field.

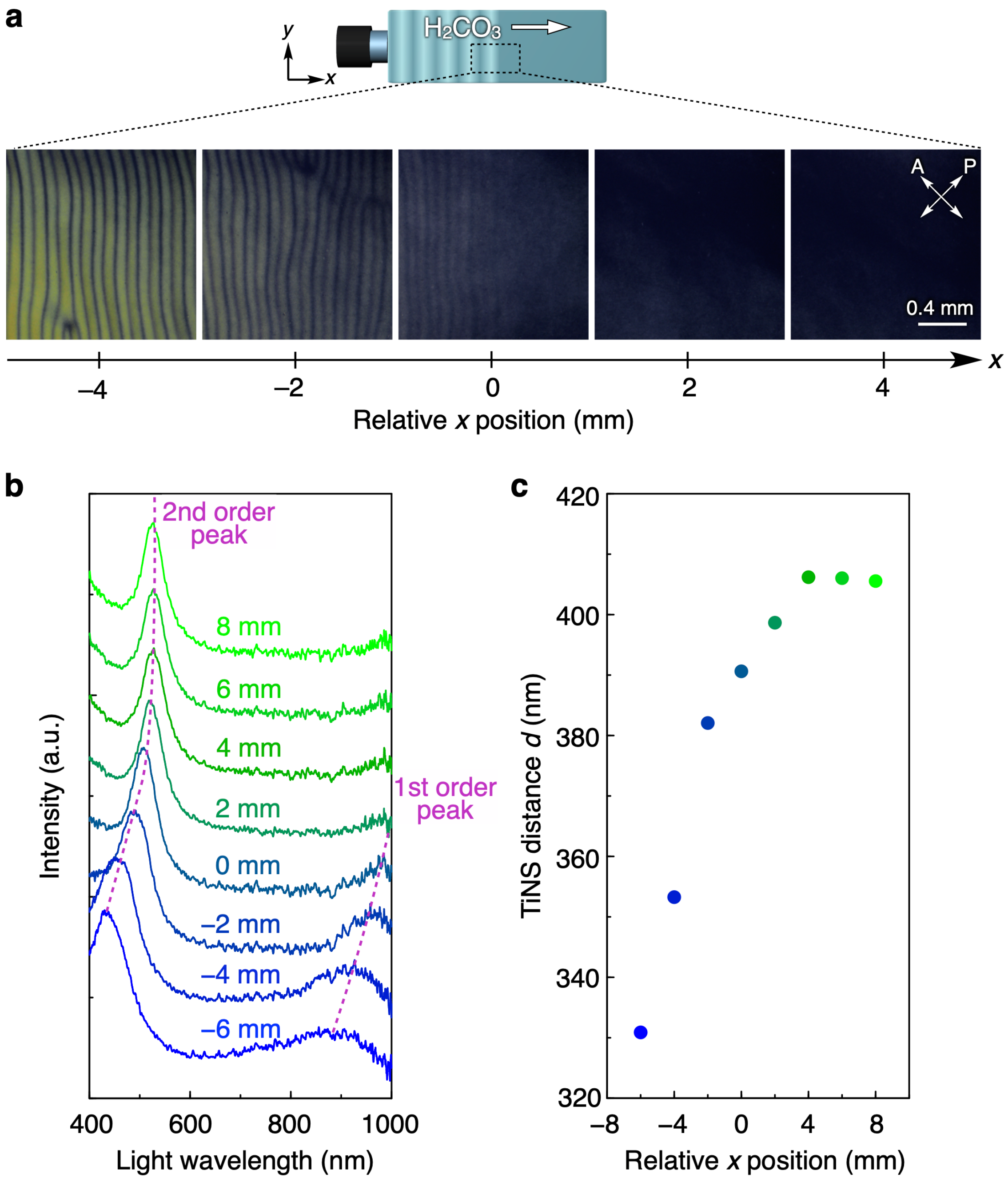

Supplementary Fig. 10 | Meso- and nanoscopic structural profiles of the front region of the propagating wave.

a, b, POM images (**a**) and microscopic Vis/NIR spectra (**b**) at various *x*-positions of the front region of the propagating wave. **c**, *x*-Position-dependent changes in the TiNS distance calculated by using the Bragg's law. A magnetically oriented TiNS dispersion ([TiNS] = 0.5 wt%) in a quartz cuvette (40 × 10 × 1 mm) was monitored at 25 °C in air (0.04% CO_2) after turning off the magnetic field. In **a**, the wavelength of the wave is almost constant regardless of the *x*-position.

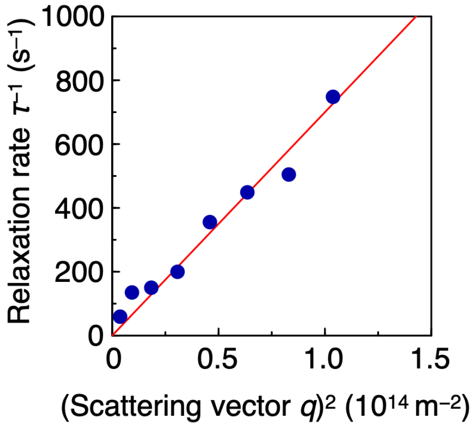

Supplementary Fig. 11 | Dynamic light scattering (DLS) measurements of an aqueous dispersion of magnetically oriented TiNSs.

Plot of the relaxation rates (inverse of a relaxation time, τ^{-1}) as a function of the square of scattering vectors q. A magnetically oriented TiNS dispersion ([TiNS] = 0.5 wt%) in a quartz cuvette (40 × 10 × 1 mm) was analyzed at 25 °C.

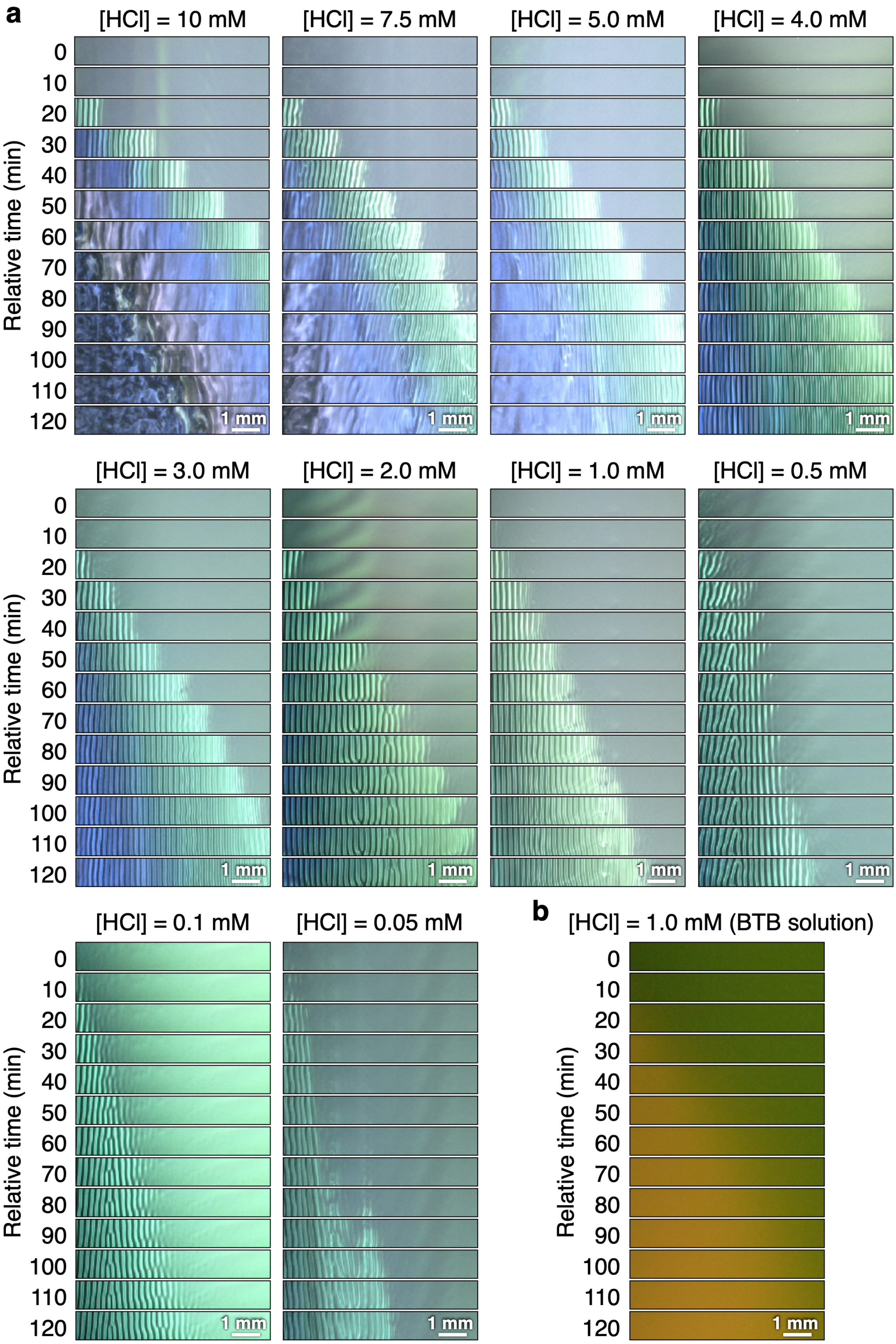
a
[HCl] = 10 mM
[HCl] = 7.5 mM
[HCl] = 5.0 mM
[HCl] = 4.0 mM
[HCl] = 3.0 mM
[HCl] = 2.0 mM
[HCl] = 1.0 mM
[HCl] = 0.5 mM
[HCl] = 0.1 mM
[HCl] = 0.05 mM
b
[HCl] = 1.0 mM (BTB solution)
Relative time (min)
0
10
20
30
40
50
60
70
80
90
100
110
120
1 mm

Supplementary Fig. 12 | Tunability of propagation velocity by the HCl concentration.

a, Magnetically oriented TiNS dispersions ([TiNS] = 0.5 wt%) in quartz cuvettes (40 × 10 × 1 mm) were treated with aqueous HCl solutions with different concentrations (0.05–10 mM) and monitored at 25 °C. Time-dependent optical images at a fixed position were shown. **b**, Time-dependent optical images of a bromothymol blue (BTB) solution after the treatment with an aqueous HCl solution ([HCl] = 1.0 mM).

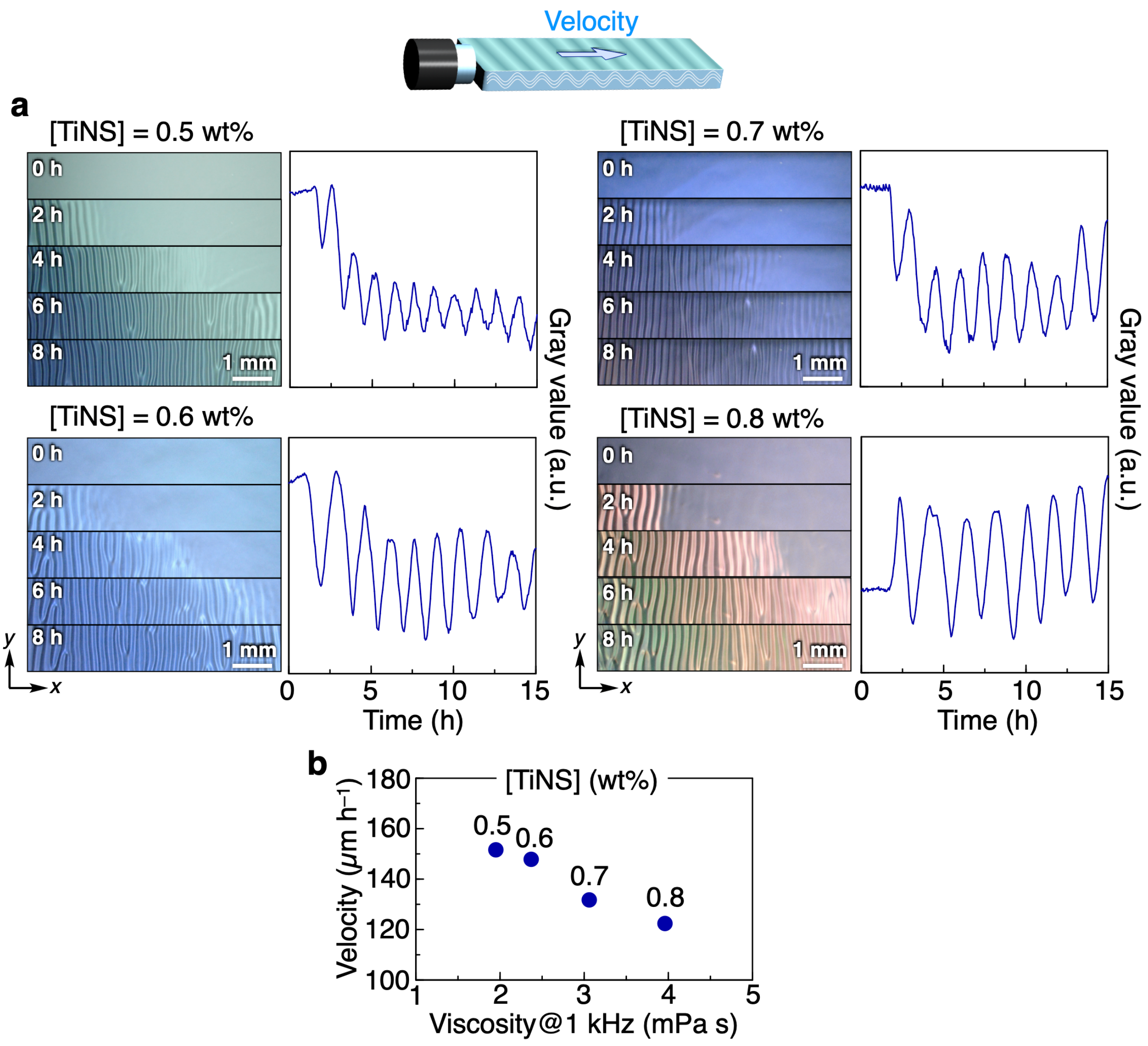

Supplementary Fig. 13 | Tunability of propagation velocity by the TiNS concentration.

a, **b**, Magnetically oriented TiNS dispersions with different concentrations (0.5–0.8 wt%) in quartz cuvettes (40 × 10 × 1 mm) were monitored at 25 °C in air (0.04% CO_2). Time-dependent optical images (left) and their gray-value profiles (right) depending on time at a fixed distance *x* (**a**) and plot of the velocity of the propagating wave as a function of the viscosity of the TiNS dispersions at a shear rate of 1 kHz (**b**).

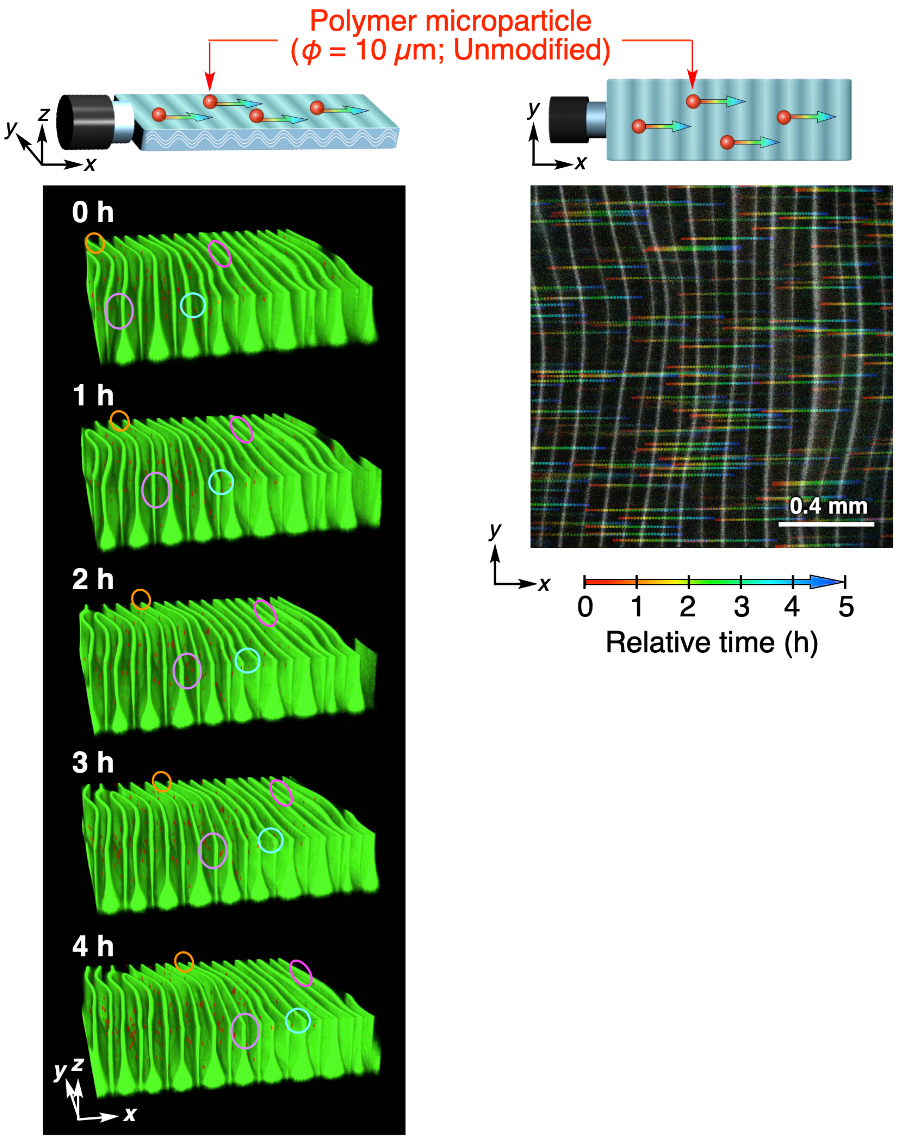

Supplementary Fig. 14 | Directional transport of unmodified microparticles by the propagating wave.

Left: Time-dependent 3D reconstructed CLSM images sectioned at z = 0.5 mm of a propagating wave (green) that transported polymer microparticles (red). A magnetically oriented TiNS dispersion ([TiNS] = 0.5 wt%) containing fluorescently labeled polymer microparticles (10 μm in diameter; unmodified) in a quartz cuvette (40 × 10 × 1 mm) was left at 25 °C in air (0.04% CO_2). 2D images were taken in a direction parallel to the xy-plane with a z-step size of 2 μm to reconstruct 3D images, where TiNSs and the microparticles were visualized by reflection (488 nm laser) and fluorescence (522 nm laser), respectively. Right: Typical trajectories of the microparticles at 10-minute intervals obtained from time-dependent 2D cross-sectional CLSM images at z = 0.5 mm.

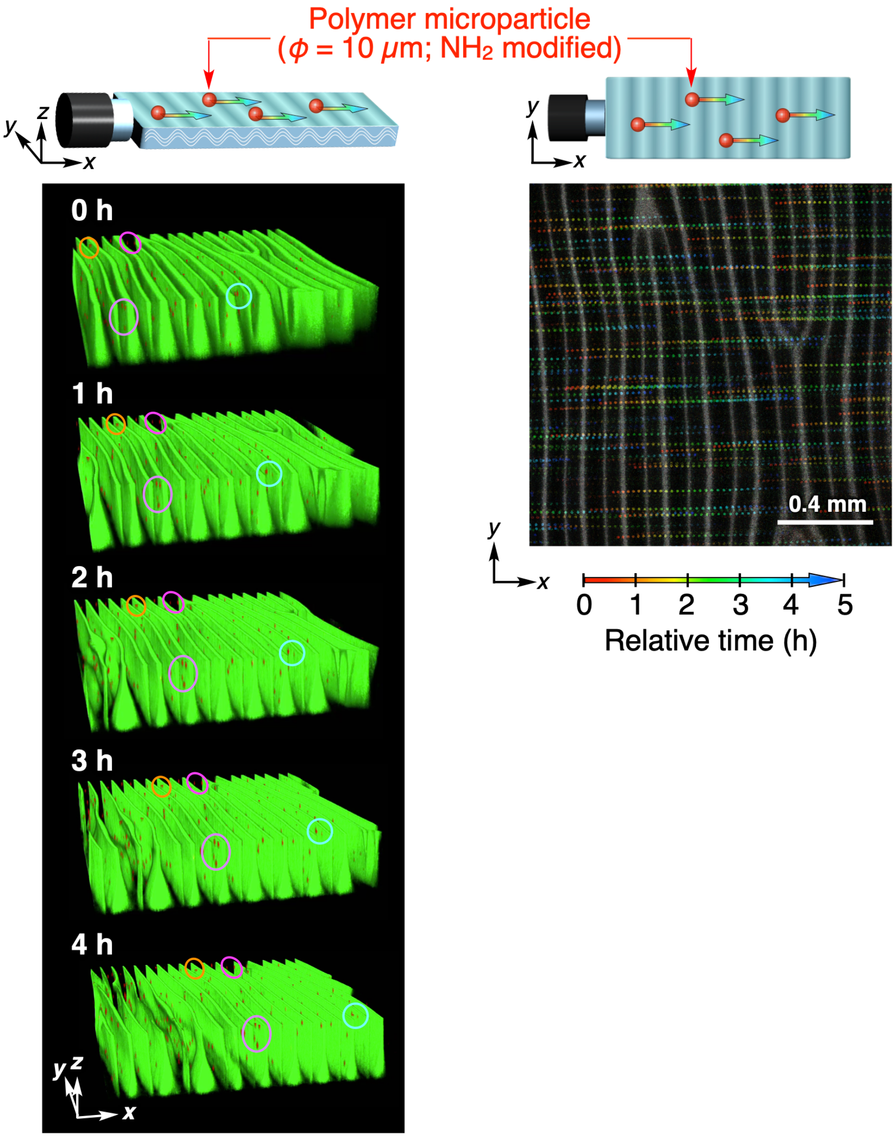

Supplementary Fig. 15 | Directional transport of NH_2 modified microparticles by the propagating wave.

Left: Time-dependent 3D reconstructed CLSM images sectioned at z = 0.5 mm of a propagating wave (green) that transported polymer microparticles (red). A magnetically oriented TiNS dispersion ([TiNS] = 0.5 wt%) containing fluorescently labeled polymer microparticles (10 μm in diameter; NH_2 modified) in a quartz cuvette (40 × 10 × 1 mm) was left at 25 °C in air (0.04% CO_2). 2D images were taken in a direction parallel to the xy-plane with a z-step size of 2 μm to reconstruct 3D images, where TiNSs and the microparticles were visualized by reflection (488 nm laser) and fluorescence (522 nm laser), respectively. Right: Typical trajectories of the microparticles at 10-minute intervals obtained from time-dependent 2D cross-sectional CLSM images at z = 0.5 mm.

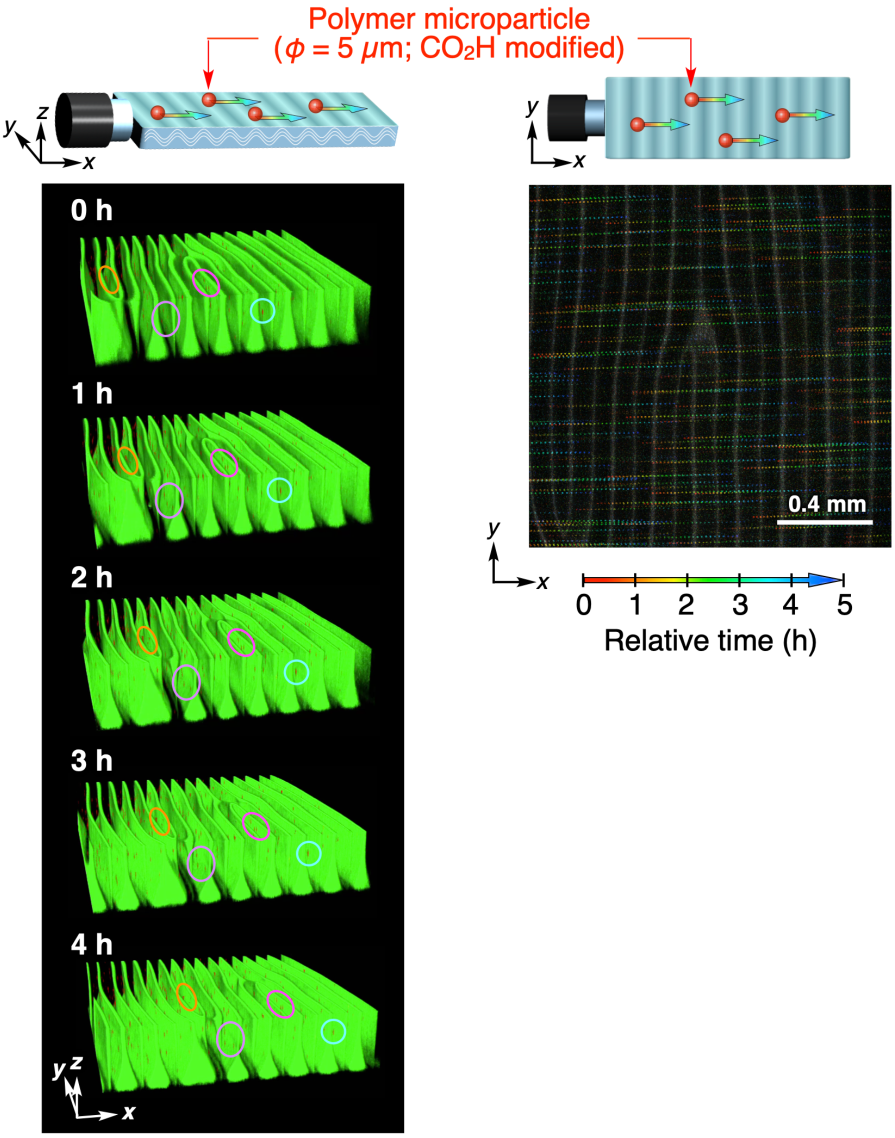

Supplementary Fig. 16 | Directional transport of 5-µm microparticles by the propagating wave. Left: Time-dependent 3D reconstructed CLSM images sectioned at z = 0.5 mm of a propagating wave (green) that transported polymer microparticles (red). A magnetically oriented TiNS dispersion ([TiNS] = 0.5 wt%) containing fluorescently labeled polymer microparticles (5 µm in diameter; CO_2H modified) in a quartz cuvette (40 × 10 × 1 mm) was left at 25 °C in air (0.04% CO_2). 2D images were taken in a direction parallel to the xy-plane with a z-step size of 2 µm to reconstruct 3D images, where TiNSs and the microparticles were visualized by reflection (488 nm laser) and fluorescence (522 nm laser), respectively. Right: Typical trajectories of the microparticles at 10-minute intervals obtained from time-dependent 2D cross-sectional CLSM images at z = 0.5 mm.

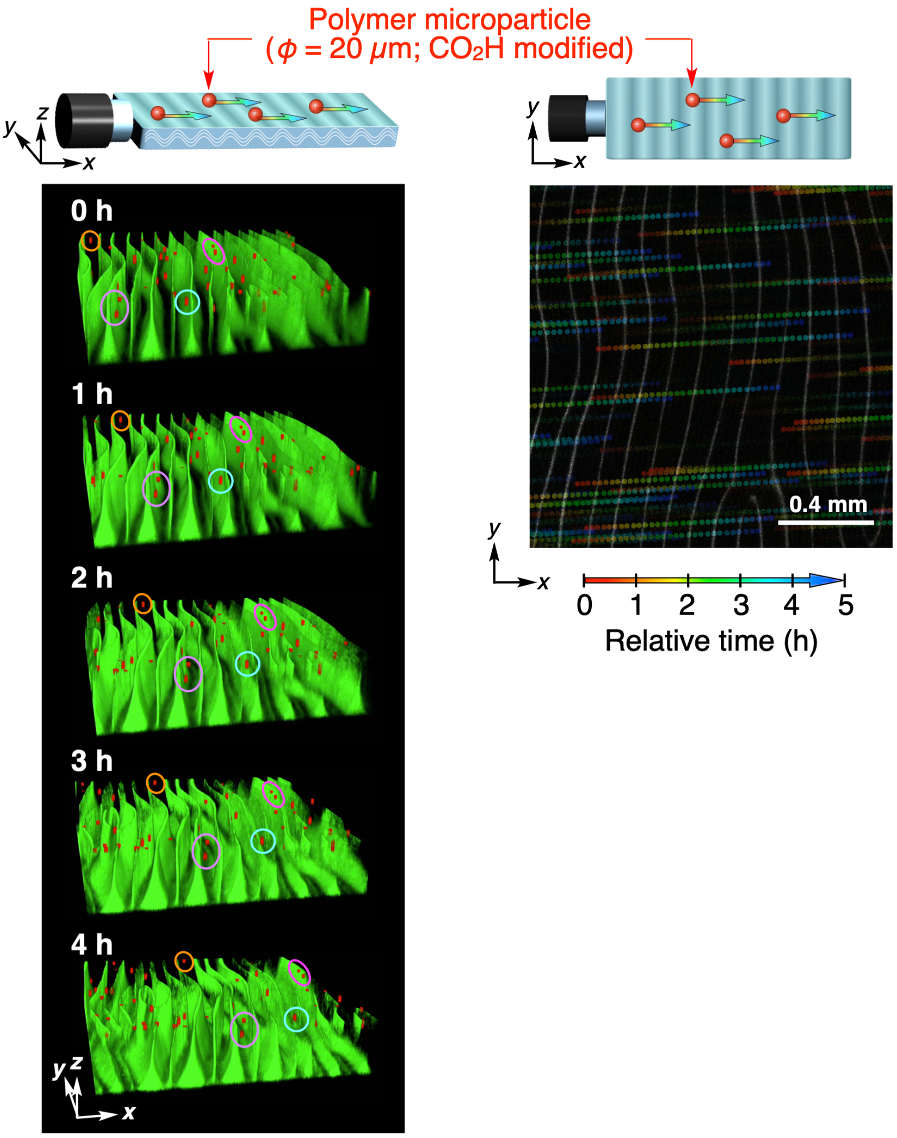

Supplementary Fig. 17 | Directional transport of 20-μm microparticles by the propagating wave. Left: Time-dependent 3D reconstructed CLSM images sectioned at z = 0.5 mm of a propagating wave (green) that transported polymer microparticles (red). A magnetically oriented TiNS dispersion ([TiNS] = 0.5 wt%) containing fluorescently labeled polymer microparticles (20 μm in diameter; CO_2H modified) in a quartz cuvette (40 × 10 × 1 mm) was left at 25 °C in air (0.04% CO_2). 2D images were taken in a direction parallel to the *xy*-plane with a *z*-step size of 2 μm to reconstruct 3D images, where TiNSs and the microparticles were visualized by reflection (488 nm laser) and fluorescence (522 nm laser), respectively. Right: Typical trajectories of the microparticles at 10-minute intervals obtained from time-dependent 2D cross-sectional CLSM images at z = 0.5 mm.

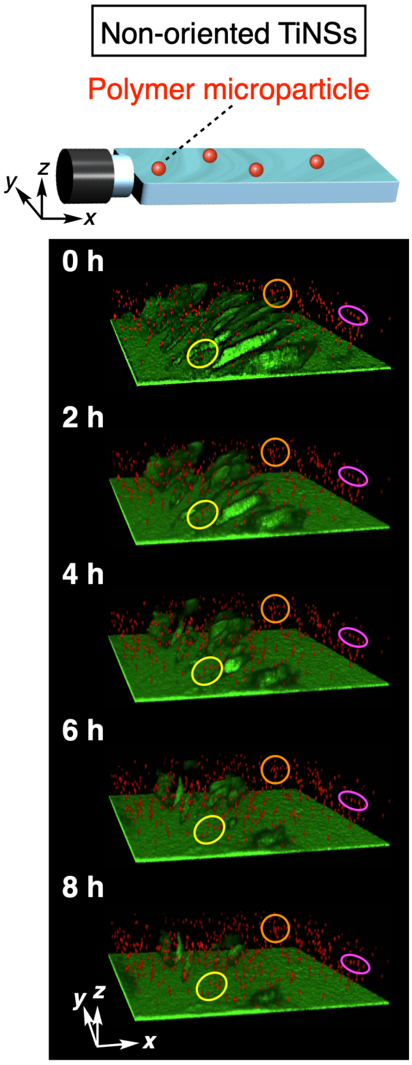

Supplementary Fig. 18 | Time-dependent behavior of a TiNS dispersion of non-oriented TiNSs. Time-dependent 3D reconstructed CLSM images sectioned at z = 0.5 mm of a non-oriented TiNS dispersion ([TiNS] = 0.5 wt%) containing fluorescently labeled polymer microparticles (10 μm in diameter; CO_2H modified) in a quartz cuvette (40 × 10 × 1 mm) at 25 °C in air (0.04% CO_2). 2D images were taken in a direction parallel to the xy-plane with a z-step size of 2 μm to reconstruct 3D images, where TiNSs and the microparticles were visualized by reflection (488 nm laser) and fluorescence (522 nm laser), respectively.
