# Supplementary material for: Propagating wave in a fluid by coherent motion of 2D colloids
Source: Nat Commun. 2021 Nov 19;12:6771. doi: 10.1038/s41467-021-26917-1 (PMC8605016; doi:10.1038/s41467-021-26917-1)
Supplement: Supplementary file 3 — Description of Additional Supplementary Files [file 41467_2021_26917_MOESM3_ESM.pdf]

Description of Additional Supplementary Files

File Name: Supplementary Movie 1
Description: **Autonomously propagating wave in air (optical microscopy).** The changes in optical images of the magnetically oriented TiNS dispersion ([TiNS] = 0.5 wt%) in a quartz cuvette (40 × 10 × 1 mm) in air (0.04% CO_2) were recorded for 20 h at 25 °C after turning off the magnetic field. The video is at 3600 times speed.

File Name: Supplementary Movie 2
Description: **No wave generation in N_2 (optical microscopy).** The changes in optical images of the magnetically oriented TiNS dispersion ([TiNS] = 0.5 wt%) in a quartz cuvette (40 × 10 × 1 mm) in N_2 were recorded for 20 h at 25 °C after turning off the magnetic field. The video is at 3600 times speed.

File Name: Supplementary Movie 3
Description: **Autonomously propagating wave in CO_2 (optical microscopy).** The changes in optical images of the magnetically oriented TiNS dispersion ([TiNS] = 0.5 wt%) in a quartz cuvette (40 × 10 × 1 mm) in CO_2 were recorded for 20 h at 25 °C after turning off the magnetic field. The video is at 600 times speed.

File Name: Supplementary Movie 4
Description: **2D profiles of the propagating wave (polarized optical microscopy).** The changes in polarized optical microscopy (POM) images under crossed Nicols of the magnetically oriented TiNS dispersion ([TiNS] = 0.5 wt%) in a quartz cuvette (40 × 10 × 1 mm) in air (0.04% CO_2) were recorded for 20 h at 25 °C after turning off the magnetic field. The video is at 3600 times speed.

File Name: Supplementary Movie 5
Description: **3D profiles of the propagating wave (confocal laser scanning microscopy).** The changes in confocal laser scanning microscopy (CLSM) images in the reflection mode of the magnetically oriented TiNS dispersion ([TiNS] = 0.5 wt%) in a quartz cuvette (40 × 10 × 1 mm) in air (0.04% CO_2) were recorded for 10 h at 25 °C after turning off the magnetic field. The whole-view and cross-sectional-view videos are sequentially shown. The videos are at 3600 times speed.

File Name: Supplementary Movie 6
Description: **3D profiles of the propagating wave that transports polymer microparticles (confocal laser scanning microscopy).** The changes in combined reflection/fluorescence confocal laser scanning microscopy (CLSM) images of the propagating wave ([TiNS] = 0.5 wt%) containing fluorescently labeled polymer microparticles (10 μm in diameter) in a quartz cuvette (40 × 10 × 1 mm) in air (0.04% CO_2) were recorded for 5 h at 25 °C. The cross-sectional-view video is shown. The video is at 3600 times speed.
